# Dr. Albert Andrew Herschler

**Published:** 1908-11

**Authors:** 


Dr. Albert Andrew Herschler, of Buffalo, died at his home in this city, October 1, 1908, aged 24 years. He graduated at the University of Buffalo, Medical Department, in 1906.

